# Epigenetic Consequences of in Utero Exposure to Rosuvastatin: Alteration of Histone Methylation Patterns in Newborn Rat Brains

**DOI:** 10.3390/ijms22073412

**Published:** 2021-03-26

**Authors:** Karolina Dulka, Melinda Szabo, Noémi Lajkó, István Belecz, Zsófia Hoyk, Karoly Gulya

**Affiliations:** 1 Department of Cell Biology and Molecular Medicine, University of Szeged, 6720 Szeged, Hungary; dulka.karolina@med.u-szeged.hu (K.D.); szabo.melinda.1@med.u-szeged.hu (M.S.); lajko.noemi@med.u-szeged.hu (N.L.); 2 Department of Medical Biology, University of Szeged, 6720 Szeged, Hungary; belecz.istvan@med.u-szeged.hu; 3 Biological Barriers Research Group, Institute of Biophysics, Biological Research Center, Eötvös Loránd Research Network, 6726 Szeged, Hungary; hoyk.zsofia@brc.hu

**Keywords:** epigenetics, H3K4me1, H3K4me3, histone methylation, immunohistochemistry, in utero, prenatal, rosuvastatin, Western blot

## Abstract

Rosuvastatin (RST) is primarily used to treat high cholesterol levels. As it has potentially harmful but not well-documented effects on embryos, RST is contraindicated during pregnancy. To demonstrate whether RST could induce molecular epigenetic events in the brains of newborn rats, pregnant mothers were treated daily with oral RST from the 11th day of pregnancy for 10 days (or until delivery). On postnatal day 1, the brains of the control and RST-treated rats were removed for Western blot or immunohistochemical analyses. Several antibodies that recognize different methylation sites for H2A, H2B, H3, and H4 histones were quantified. Analyses of cell-type-specific markers in the newborn brains demonstrated that prenatal RST administration did not affect the composition and cell type ratios as compared to the controls. Prenatal RST administration did, however, induce a general, nonsignificant increase in H2AK118me1, H2BK5me1, H3, H3K9me3, H3K27me3, H3K36me2, H4, H4K20me2, and H4K20me3 levels, compared to the controls. Moreover, significant changes were detected in the number of H3K4me1 and H3K4me3 sites (134.3% ± 19.2% and 127.8% ± 8.5% of the controls, respectively), which are generally recognized as transcriptional activators. Fluorescent/confocal immunohistochemistry for cell-type-specific markers and histone methylation marks on tissue sections indicated that most of the increase at these sites belonged to neuronal cell nuclei. Thus, prenatal RST treatment induces epigenetic changes that could affect neuronal differentiation and development.

## 1. Introduction

Statins (3-hydroxy-3-methylglutaryl coenzyme A (HMG-CoA) reductase inhibitors) are a class of lipid-lowering agents used in the treatment of high cholesterol levels [1]. Apart from the inhibition of cholesterol synthesis, reduction of levels of low-density lipoproteins and triglycerides, and stimulation of the expression of high-density lipoproteins, they also strongly modulate the inflammatory cells surrounding atherosclerotic plaques [2,3]. Statins may also have beneficial effects on the central nervous system (CNS) [4,5,6]. In vitro [7] and animal studies [8,9] have demonstrated that statins can attenuate neuroinflammation. Furthermore, statins have long been known to induce apoptosis in various cancer cell lines [10,11], and are associated with antitumor properties [12,13].

Besides their beneficial properties, statins also have numerous adverse effects [14]. As a result of their potentially harmful, but not well documented effects on the embryo, it is recommended that statin treatment be discontinued three months before attempting to become pregnant and that statins should not be used during pregnancy or breastfeeding [15] because of the potential risk of fetal abnormality [16]. Some malformations were reported in fetuses after pregnant mothers were exposed to lipophilic statins (such as simvastatin, lovastatin, atorvastatin, cerivastatin, and fluvastatin) as compared to hydrophilic statins (pravastatin and rosuvastatin (RST)) [17,18]. For example, in a prospective, observational cohort study, Taguchi et al. [19] found no evidence of major malformations, spontaneous or therapeutic abortions, or stillbirths, but noted a younger gestational age at birth and lower birth weights from patients using RST. Animal models provided some evidence for the teratogenic effects of lipophilic statins on pregnancy outcomes, including decreased fetal body weight and survival rate, and unusual neonatal development [20,21,22].

RST, one of the highest-selling prescription drugs on the market (Crestor; AstraZeneca Pharmaceuticals, LP, Wilmington, DE, USA), exhibits the greatest inhibitory effect on cholesterol biosynthesis [23], and among the statins, it alters the high-density lipoprotein profile most favorably [24]. Only small amounts of RST have been shown to pass into breast milk [25]. In in vitro studies, RST strongly inhibited the expression of certain pro-inflammatory genes, while concomitantly vigorously stimulating several anti-inflammatory genes [7]. Its beneficial effects on the expression of inflammasome-related genes in animal models [26] or humans [27] have also been noted.

Histone modification is one of the main mechanisms of epigenetic modification regulating gene expression. This modification requires several different histone-modifying enzymes, including “writers”, which attach modifications to histone tails, “erasers”, which remove modifications, and “readers”, which recognize these modifications [28,29,30,31]. The N-terminal tails of histones are subject to a number of highly site- and residue-specific posttranslational modifications, including methylation, acetylation, phosphorylation, ubiquitylation, and SUMOylation, that are implicated in influencing gene expression and genome function, as they coordinate the recruitment of chromatin remodelers and the transcriptional machinery for transcriptional regulation [32]. Of all the known modifications, Lys (K) histone methylation has been regarded as a stable chromatin modification that, together with DNA methylation, defines epigenetic programs [33]. Lys histone methylation has been linked to transcription initiation and elongation and heterochromatin silencing, among other mechanisms. Lys methylation marks, such as H3K4 and H3K36, are implicated in the activation of transcription and are linked to open chromatin, while the H3K9, H3K27, and the H4K20 Lys methylation sites are associated with transcriptional repression, as is characteristic of condensed chromatin [33,34]. The complexity of the methylation patterns of histone proteins and the methylation state at any given Lys residue (unmethylated, mono- (me1), di- (me2), or trimethylated (me3)) further influence gene expression.

As recent studies have emphasized the importance of maternal effects on chromatin structure and the interrelationship between the genome, epigenome, and environment [35,36], our aim was to investigate whether RST elicited molecular epigenetic events such as histone methylation in the brains of the newborn rats whose mothers had been treated chronically with the drug.

## 2. Results

### 2.1. Histone Methylation Patterns of Absolute Controls and Vehicle-Treated Controls Did Not Differ Significantly

**Figure 1:**
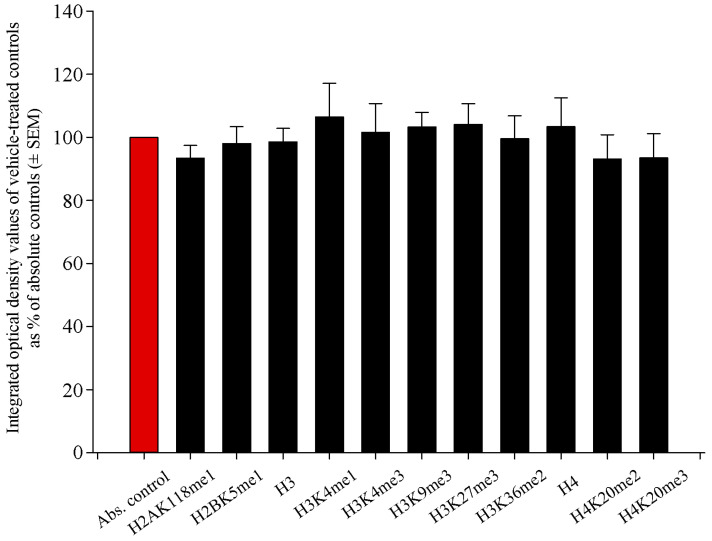
Quantitative Western blot analyses of histone methylation patterns in the brains of absolute control and vehicle-treated newborn rats. Protein contents from the absolute and vehicle-treated controls were determined and assayed on Western blots. Gel-loaded protein samples were separated by gel electrophoresis, transferred to nitrocellulose membranes, and assayed for immunoreactivity with antibodies that recognize core histones and histone methylations. Grayscale digital images of the immunoblots were scanned and processed under identical settings to allow comparisons between Western blots from different samples. Values indicate the means ± SEM from at least 5 separate experiments. Integrated optical density data were analyzed by ANOVA using SigmaPlot. No statistically significant differences were observed between the absolute and vehicle-control samples. Hence, we refer to vehicle-treated controls hereafter as simply “controls”, and further data presentation uses the vehicle-based controls as the reference point.

When investigating epigenetic events in the newborn brain after the administration of RST, we assumed that liver pâté, the vehicle used to deliver RST, would not elicit changes in histone methylation patterns. To reveal such possible effects of the liver pâté, we assayed H2AK118me1, H2BK5me1, H3, H3K4me1, H3K4me3, H3K9me3, H3K27me3, H3K36me2, H4, H4K20me2, and H4K20me3 levels using Western blots from absolute control and vehicle-treated control newborn rat brain samples. Our data showed that liver pâté, as a nutritional supplement, had no significant effect on the methylation patterns of these sites (Figure 1). As all vehicle-based controls had values between 93.2% ± 7.7% and 106.4% ± 10.7% of the absolute controls, with no significant differences among them across at least five separate experiments, we refer to vehicle-treated controls hereafter as merely “controls”, and further data presentation uses the vehicle-based controls as a reference point.

### 2.2. Prenatal RST Exposure Does Not Affect Cell Composition in the Newborn Brain

**Figure 2:**
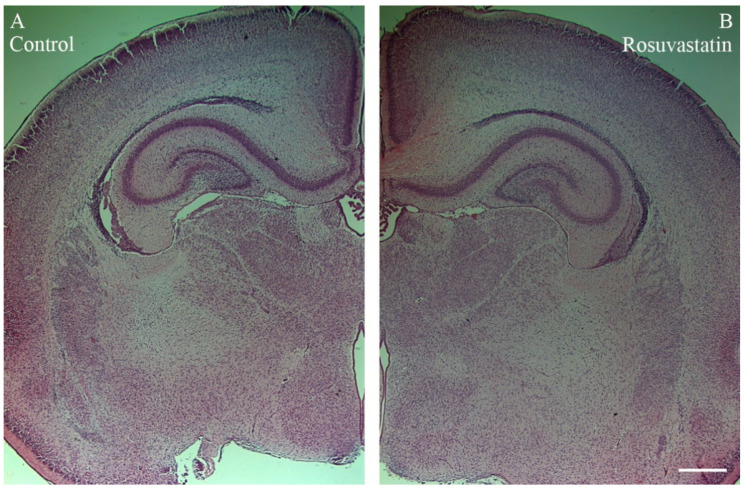
Histological architecture of the newborn rat brain. No differences were observed in paraffin-embedded, H&E-stained tissue sections of control (**A**) and rosuvastatin (RST)-treated newborn (**B**) brains by light microscopy. Scale bar: 1 mm.

**Figure 3:**
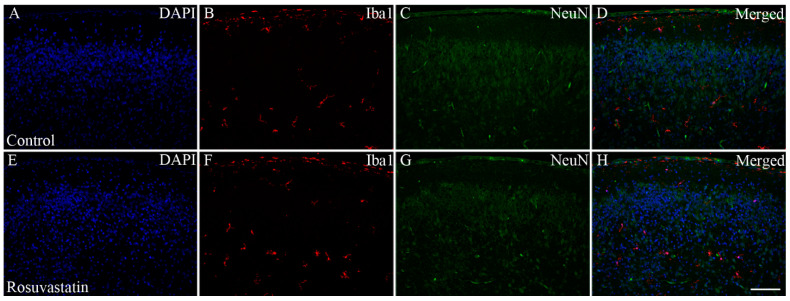
Double immunofluorescent staining for Iba1 and NeuN. Microglia were labeled with the anti-Iba1 antibody/Alexa Fluor 568 goat anti-rabbit IgG complex (red), whereas neurons were detected with the anti-NeuN antibody/Alexa Fluor 488 goat anti-mouse IgG complex (green). Cell nuclei were labeled with DAPI (blue). Representative pictures with lower (20×) magnification show that prenatal exposure to RST did not cause abnormalities in the cytoarchitecture by light microscopy. The ratio of microglia (**B**,**F**) to neurons (**C**,**G**) is similar between the control (**A**–**D**) and RST-treated (**E**–**H**) newborn brains. Scale bar: 100 µm.

**Figure 4:**
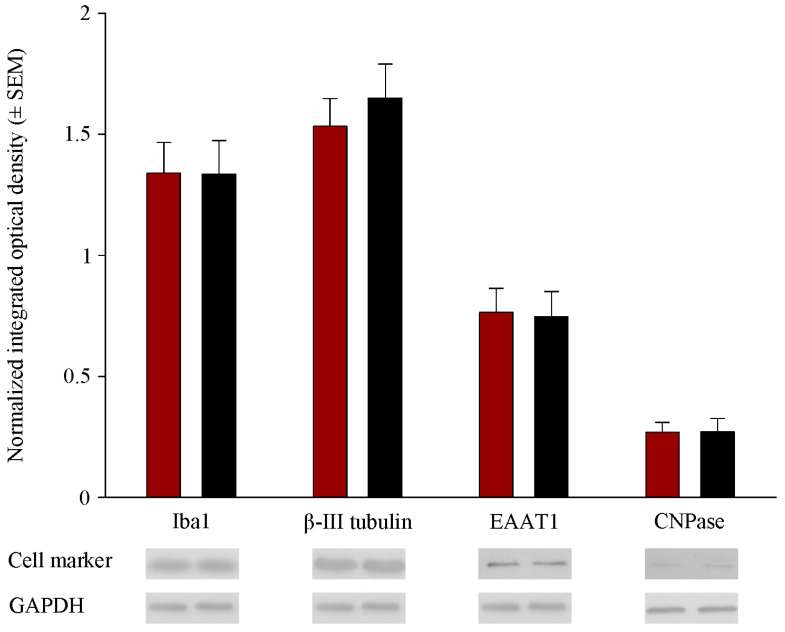
Quantitative Western blot analyses of the main cell types from control and RST-treated newborn rat brains. Controls (red column) and RST-treated (black column) samples were analyzed quantitatively on Western blots for microglial, neuronal, astrocyte, and oligodendrocyte markers. Protein samples (15–30 μg) were separated by gel electrophoresis, transferred to nitrocellulose membranes, and assayed for reactivity toward the Iba1 (microglia marker), beta III tubulin (neuron-specific marker), EAAT1 (astrocyte marker), CNPase (oligodendrocyte marker), and GAPDH proteins. Error bars indicate integrated optical density values (means ± SEM from at least 5 separate experiments) normalized to the values of the internal standard GAPDH. Representative Western blot pictures are shown below the graphs. No statistically significant differences were found between the control and the RST-treated groups.

**Figure 5:**
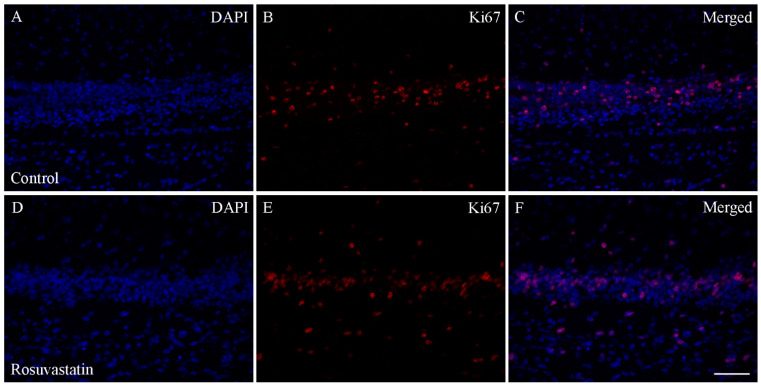
Ki67 immunohistochemistry of the hippocampus in control and prenatally RST-exposed newborn rats. The expression of the Ki67 protein in the tissue areas was quantified by the percentage of Ki67-positive nuclei (red) over the total number of nuclei (positive and negative nuclei). Immunohistochemical data analysis detected highly similar incidences for Ki67-immunopositive cells (**B**,**E**) among the DAPI dye-labeled cell nuclei (blue; **A**,**D**) for both the controls (12.9%) and the prenatally RST-treated rats (12.5%). Merged pictures (**C**,**F**) are shown. Scale bar: 50 µm.

RST exposure in utero did not cause structural abnormalities in the newborn brain at the level of light microscopy as evidenced by H&E staining (Figure 2). Double immunofluorescent staining for microglial and neuronal cell markers revealed that the ratios of these cells did not change between the controls and the RST-treated groups (Figure 3). Quantitative Western blot analysis of cell-specific markers corroborated that prenatal exposure to RST did not cause abnormalities in cell composition in newborn brains, as the ratios of these cells did not change significantly between the control and treated groups (Figure 4). These observations were supported by Ki67 fluorescent immunohistochemistry, as proliferation rates in both the control and prenatally RST-exposed rat brains were in the same range (Figure 5). Analyses of the immunohistochemical data detected approximately the same incidence of Ki67-immunopositive cells among the DAPI-labeled cell nuclei of both control and prenatally RST-treated rats. Of a total of 4268 cells analyzed from the controls and 3557 cells analyzed from RST-treated newborns, 554 (12.9%) and 448 (12.5%) cells were Ki67-positive, respectively.

### 2.3. In Utero RST Exposure Alters Histone Methylation Patterns in the Newborn Brain

**Figure 6:**
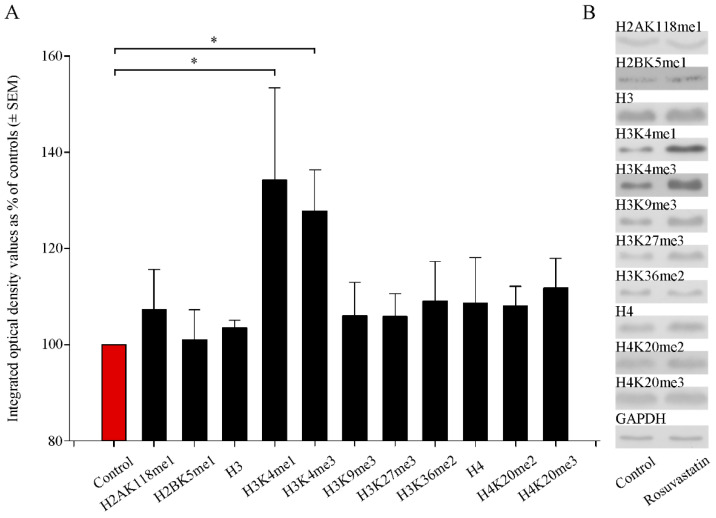
Quantitative Western blot analyses of histone methylation patterns in newborn rat brains. (**A**) Protein contents from the vehicle-treated controls and RST-treated newborns were determined and assayed by Western blot. Gel-loaded protein samples were separated by gel electrophoresis, transferred to nitrocellulose membranes, and assayed for immunoreactivity using antibodies that recognize different histone methylations. Grayscale digital images of the immunoblots were scanned and processed at identical settings to allow comparisons between Western blots from different samples. Integrated optical density values were calculated as the percentage of the vehicle-treated control values that had been normalized to the internal standard GAPDH. Prenatal RST exposure induced a general increase in the Lys-methylated sites of several histone proteins. Quantitative analysis showed that H3 histone mono- and tri-methylation at Lys 4 (H3K4me1 and H3K4me3) increased significantly (134.3% and 127.8% of vehicle-treated controls, respectively). (**B**) Representative Western blot images of immunoreactivity toward antibodies specific to the methylation sites and states, together with the GAPDH immunoreactive bands that served as the internal standards. Data were analyzed with the Mann–Whitney rank–sum test. Values are presented as the means ± SEM from at least 5 separate experiments, where *p* < 0.05 is considered significant. (* *p* < 0.05).

Several antibodies that recognize different methylation sites for the H2A, H2B, H3, and H4 histones were used in the Western blotting experiments (Table S2). We found that prenatal RST treatment induced a general, nonsignificant increase in H2AK118me1, H2BK5me1, H3, H3K9me3, H3K27me3, H3K36me2, H4, H4K20me2, and H4K20me3 levels, to 101.0%–111.7% of the control levels (Figure 6). However, the levels of H3 histone mono- and tri-methylation at Lys 4 (H3K4me1 and H3K4me3) were elevated significantly (134.3% ± 19.2% and 127.8% ± 8.5%, respectively) compared to the control values. These modifications are known to play roles in transcription activation.

### 2.4. The Increase in H3K4me1 and H3K4me3 Is Localized Mainly to Neuronal Cell Nuclei

**Figure 7:**
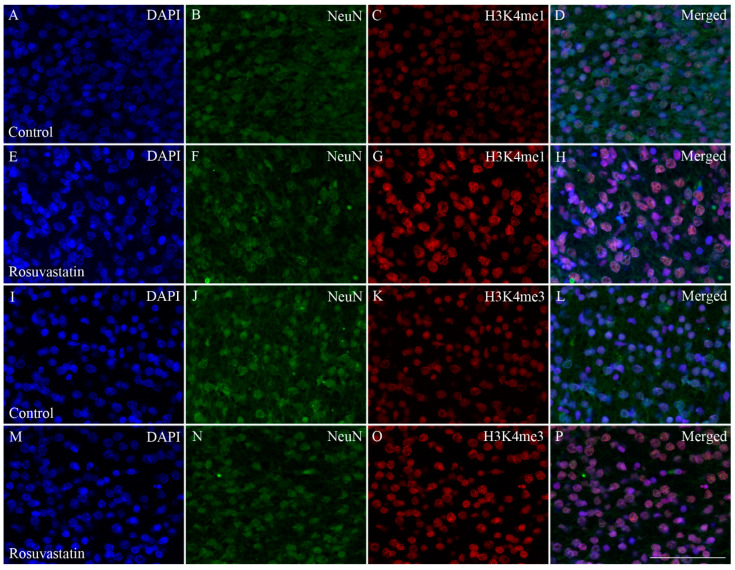
Colocalization of H3K4 methylation patterns and NeuN immunoreactivity in neurons of the newborn brain. DAPI-labeled cell nuclei are shown in blue (**A**,**E**,**I**,**M**). Immunoreactivity of the neuron-specific marker NeuN (**B**,**F**,**J**,**N**; shown here in green) and the histone methylation markers H3K4me1 and H3K4me3 (**C**,**G**,**K**,**O**, respectively; shown here in red) were colocalized in merged images (**D**,**H**,**L**,**P**). These immunohistochemical data corroborated the results of the Western blot analyses, as the immunofluorescent signals of the methylation marks were more intense in the sections from the RST-exposed newborns (**G**,**O**) as compared to the controls (**C**,**K**). Scale bar: 75 μm.

**Figure 8:**
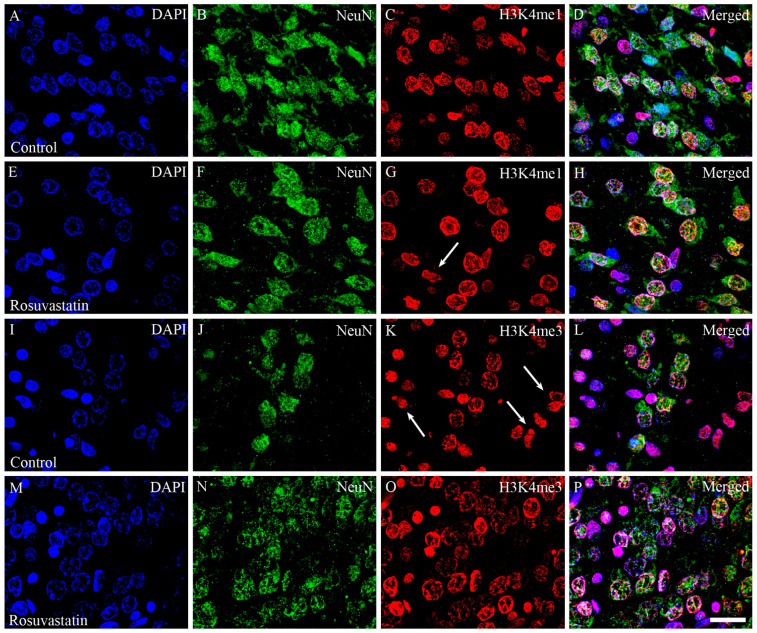
Laser confocal microscopy images of H3K4me1 and H3K4me3 immunoreactivities in neuronal nuclei. Paraffin sections of brains from the control (**A**–**D**,**I**–**L**) and RST-treated newborn rats (**E**–**H**,**M**–**P**) were immunostained for NeuN (shown here in green) and H3K4me1 or H3K4me3 (shown here in red), then visualized by confocal microscopy. Cell nuclei were labeled with DAPI (blue). Note the overwhelmingly neuronal localization of the histone methylation marks. Some NeuN-negative non-neuronal cells, notably in panels (**G**,**K**) (white arrows), also display H3K4me1 and H3K4me3 marks. Scale bar: 20 μm.

**Figure 9:**
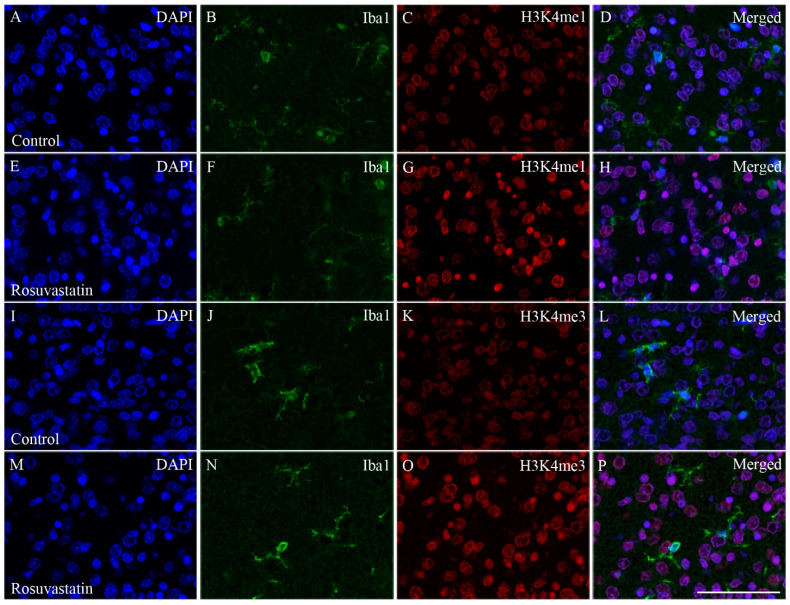
Colocalization of H3K4 methylation patterns and Iba1 immunoreactivity in the microglial cells of the newborn brain. In a newborn rat brain, the majority of the cells are neurons. At birth, microglia are unevenly distributed and located in specific “hotspots” such as the ventricular zone. Due to their overwhelmingly neuronal localization, H3K4me1 and H3K4me3 immunoreactivities (shown here in red) were more intense in the sections cut from the RST-treated newborns (**E**–**H**,**M**–**P**) as compared to the controls (**A**–**D**,**I**–**L**). A few H3K4 methylated cell nuclei belonged to Iba1-positive microglia (shown here in green). DAPI-labeled nuclei are shown in blue (**A**,**E**,**I**,**M**). Scale: 75 μm.

**Figure 10:**
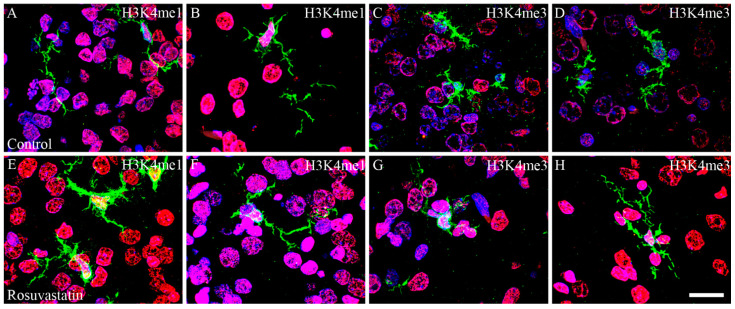
Laser confocal microscopy images of H3K4me1 and H3K4me3 immunoreactivities in microglial nuclei. Newborn brain sections from the control (**A**–**D**) and RST-treated rats (**E**–**H**) were immunostained for Iba1 (green), and H3K4me1 or H3K4me3 (red), then visualized by confocal microscopy. DAPI-labeled cell nuclei are blue. Note in the merged pictures the very few microglia that colocalize with the histone methylation marks. Scale bar: 20 μm.

**Figure 11:**
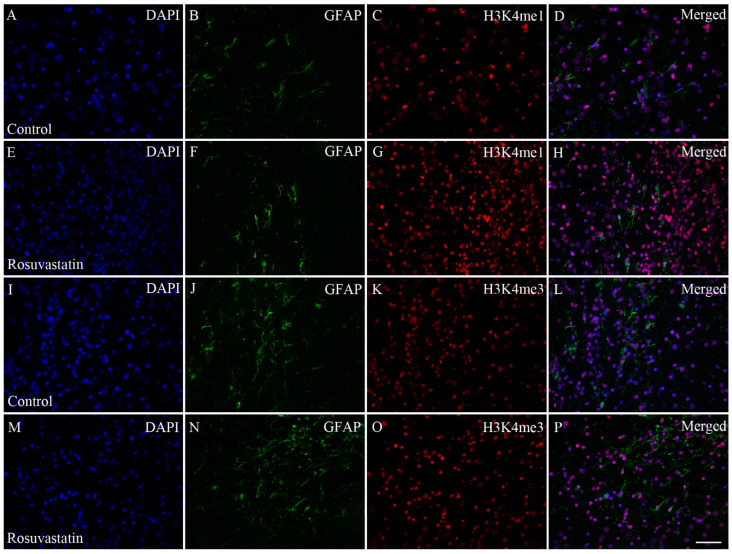
Double immunofluorescence staining for H3K4me1 and H3K4me3 in GFAP-positive astrocytes. In the neonatal rat brains, GFAP-positive cells were few, and unevenly distributed (green; **B**,**F**,**J**,**N**). DAPI-labeled cell nuclei are blue (**A**,**E**,**I**,**M**), and H3K4me1 (**C**,**G**) and H3K4me3 (**K**,**O**) marks are shown here in red. Merged pictures show (**D**,**H**,**L**,**P**) that few GFAP-positive astrocytes exhibited H3K4me1 and H3K4me3 marks. Scale bar: 50 μm.

Cell-specific markers were used to localize the increases in H3K4me1- and -me3-immunopositivity in the cerebral tissue sections. Most of the immunoreactivity was localized to NeuN-positive neuronal cell nuclei, which constitute the vast majority of the parenchyma, and typically displayed the large nuclei of neuronal morphology (Figure 7 and Figure 8). Interestingly, besides neurons, few Iba1-positive microglia (Figure 9 and Figure 10) and GFAP-positive astrocytes (Figure 11) exhibited increased H3K4me1and H3K4me3 immunopositive signals, although the numbers of these cells in the neonatal brain were negligible. These glial cells displayed smaller nuclei with more compact chromatin, and they were smaller than the neurons in the newborn rat brain. Hardly any CNPase-positive oligodendrocytes were detected (data not shown), as myelogenesis occurs predominantly postnatally.

## 3. Discussion

Although the beneficial effects of statins are well documented [1], there are some circumstances, including pregnancy, in which their use requires caution. For example, HMG-CoA reductase activity is required for normal placental development in mammals. The inhibition of this enzyme by statins may disrupt membrane synthesis, cellular proliferation and growth, and metabolism and protein glycosylation, which are crucial for the normal development of the placenta [17]. Limited data exist on the effect of statins in pregnancy, and there is no specific pattern of congenital anomalies associated with statin use [18]. Exposure to lipophilic statins is hypothesized to be of greater risk to the fetus than hydrophilic statins, because of their greater ability to reach the fetus in larger concentrations as a result of placental transport [17]. Studies have occasionally reported beneficial effects from statin use; for example, Elahi et al. [37] demonstrated that statin treatment in hypercholesterolemic pregnant mice reduced certain cardiovascular risk factors in their offspring. Statins also proved beneficial in preventing preeclampsia, thus ameliorating the risks of structural abnormalities to the fetus [18,38]. However, the use of statins in human pregnancy is currently not recommended, because of the potential teratogenic effects observed in animal experiments on one hand, and as a precaution owing to the lack of data supporting an indication for their use in pregnancy on the other. Indeed, animal models (rats, mice, rabbits) provide sporadic evidence for the teratogenicity of statins on pregnancy outcomes [21,22,39].

The nervous system comprises several different cell types that are defined by morphology, function, anatomical location, and specific patterns of gene expression. The establishment of these complex and highly regulated cell fates requires the spatial and temporal coordination of gene transcription in the proper order, number, and location. In the developing brain, neurons are generated first, followed by the supporting glia. Lineage studies indicate that the developing brain contains multipotent progenitor cells capable of generating both neurons and glia, and that cell fate restriction may be a consequence of a series of gene expression events and extracellular signals acting on multipotent progenitors [40]. 

On postnatal day 1, the newborn rat brain is a tissue populated mostly by neurons that are complemented gradually with non-neuronal cells. Bandeira et al. [41] reported that > 90% of the cells are neurons at this stage, and only about 6% of the cells had non-neuronal phenotypes. They also showed that the neuronal population initially present at birth was further reduced by apoptosis and that the number of non-neuronal, mostly glial, cells increased. Neurons and glial cells then worked together to achieve proper neuronal development and normal brain function. Microglia, the resident immune cells of the CNS, infiltrate the embryonic brain early and develop side by side with the neurons. At this early stage, when the first wave of synaptogenesis occurs, microglia are the only major non-neuronal cell type present in the CNS [42]. The generation of astrocytes and oligodendrocytes occurs in a temporally distinct, yet overlapping, pattern. In rats, neurogenesis peaks at embryonic day 14, astrocytogenesis at postnatal day 2 (they are produced largely during the final stage of neurogenesis), and oligodendrocytogenesis at postnatal day 14, although oligodendrocyte precursors appear somewhat earlier [43].

Mammalian embryonic development and subsequent fetal development involve precise molecular interactions between intrinsic factors such as genetics and epigenetics, and extrinsic maternal factors, such as environmental perturbations, drugs, or even maternal nutrition, as nutritional components that could influence the epigenetic landscape in the fetus and thus developmental processes [44]. Euchromatin allows transcription factors to interact with gene promoters and activate lineage-specific genes, while heterochromatin remains inaccessible to transcriptional activation [32]. During embryonal development, the histone methylation landscape of brain cells is sensitive to a wide range of environmental cues. Diet is now recognized as a major environmental factor that may contribute to controlling the physiological and pathophysiological aspects of homeostasis, metabolism, and gene expression [45,46]. It was therefore important to rule out any interference of liver pâté, as the vehicle for RST, on epigenetic mechanisms. We ruled out potential impacts of this diet supplement on the histone methylation patterns, as they did not significantly change their levels between the absolute and vehicle-treated control newborn rat brains. We did not find any signs of RST histotoxicity or changes in the cellular compositions of newborn brains through light microscopy. It is important to note that most of the histone methylation marks we detected in this study were localized to neurons, and to a much smaller extent, to microglia. Astrocytes and oligodendrocytes develop and populate the newborn brain at later time points.

The dysregulation of chromatin decreases viability and normal cell functions and leads to various neurodevelopmental and psychiatric diseases [47]. H3K4 methylation has been observed in genes that are important for the regulation of cell differentiation, proliferation, and apoptosis [48]; for example, homeobox genes, known for their role in embryonic development, are regulated by H3K4 methylation [49]. H3K4me3 is almost always associated with RNA polymerase II occupancy of the promoters at the sites of active gene expression [50]. H3K4me1 and H3K4me3 marks are commonly located in euchromatin, are broadly associated with transcriptional regulation and the epigenetic tagging of promoters and enhancer sequences, and are known to allow the DNA to adopt a more “open” conformation and recruit chromatin-modifying factors [51]. The proper regulation of H3K4 methylation is pivotal for healthy brain development, as mutations associated with the loss and gain of H3K4 methylation could potentially result in intellectual disability, autism, microcephaly, seizure disorders, and other neurological diseases in early childhood [47]. It is possible that this epigenetic mark is involved more broadly in the pathophysiology of some neurodevelopmental disorders, as multiple regulators of H3K4 methylation are associated with neurodevelopmental diseases [52,53]. There can be little doubt that the H3K4 methylation landscape of brain cells is sensitive to a wide range of environmental perturbations [47]; indeed, the H3K4me3 mark undergoes global and gene-specific alterations in the hippocampus of fear-conditioned animals. Gupta et al. [54] found that H3K4me3 was upregulated in the rat hippocampus 1 h after contextual fear conditioning and that this increase was reversible. Furthermore, the activation of the maternal immune system by the viral mimic polyriboinosinic¬–polyribocytidilic acid, which leads to behavioral deficits in the adult offspring, could result in robust but transient changes in H3K4 methylation at the genes encoding cytokines and other signaling molecules in the fetal brain [55]. Prenatal exposure to the alkylating and antimitotic agent methylazoxymethanol, which leads to several anatomical and behavioral abnormalities in adulthood similar to those observed in patients with schizophrenia, resulted in decreased H3K4 methylation in the adult prefrontal cortex [56].

In this study, we focused on methylations of the Lys (K) residues of the four core histones. These epigenetic modifications, particularly in the case of the histone H3, are involved in a wide range of biological processes, including the activation and repression of transcription; nevertheless, one methyl mark by itself might still have a limited biological message. As mentioned above, H3K4me is a mark that, on a genome-wide scale, is associated broadly with transcriptional activation and the epigenetic tagging of promoters and enhancers [51]. Hence, H3K4me1/3 sites correlate positively with gene expression similarly to H3K36 methylation. The methylation of histone H3 lysine 36 (H3K36) plays an important role in the partitioning of chromatin into distinctive domains, as well as in the regulation of a wide range of biological processes [57,58]. H3K36me2 regulates the distribution of H3K27me3 [57], a hallmark of heterochromatin. A study on the epigenetic silencing of inflammation-related genes demonstrated that repressive histone marks (e.g., H3K27me3) correlated with a silencing of their expression [59]. Similar to H3K27, the methylation of H3K9 was shown to be involved in heterochromatin formation and transcriptional silencing [60]. H3K9me3 is a typical mark of constitutive heterochromatin, while H3K27me3 is usually enriched in facultative heterochromatin [61]. H3K9me3 modification is associated with changes in gene transcription by alterations to the chromatin structure. Another hallmark of heterochromatin is a strong enrichment of H4K20me2/3; in fact, H3K9me3 is required for the induction of H4K20me2/3 [61]. Hence, the H4K20 histone methyl mark is associated with gene repression [34]. Besides H3 and H4, there are only a few known methylation marks in H2A and H2B; for example, Barski et al. [51] reported that H2BK5me1 is an activation mark associated with the active promoters downstream of transcription start sites. Interestingly, no data are available on the Lys methylation of H2A, particularly of H2AK118.

In this study, we provided evidence that, in newborn rat brains, prenatal exposure to RST alters the methylation landscape in general, and the H3K4me1 and H3K4me3 histone modifications in particular. We observed that RST significantly elevated the levels of H3K4me1 and H3K4me3 compared to the controls. Furthermore, we did not find any histological structural alteration in newborn brains that could be related to RST treatment. It remains to be determined in future studies whether the changes observed in the H3K4 methylation patterns are: (1) linked to specific loci or are genome-wide, (2) reflect an adaptive or maladaptive response to RST, or (3) are the outcomes of secondary or tertiary processes in response to RST treatment. The identification of the genomic sequences involved in the control of the embryonic development of offspring whose mothers had been treated with RST remains a formidable challenge. Interestingly, statins, in general, did not directly inhibit the activity of the major epigenetic modifying enzymes, such as, “writers” or “readers” [62]. Since 50% of all pregnancies are unplanned [63], the possibility exists that a pregnant woman may be taking a statin at least for a while. The epigenetic changes elicited by rosuvastatin are not good or bad unless definitely proven either way. Although there are no such data available yet on humans, our data could provide a warning. Prenatal statin therapies, therefore, require caution and warrant further investigation until a more detailed picture of their effects on the epigenetic spectrum emerges.

## 4. Materials and Methods

### 4.1. Animal Handling and Treatment

Pregnant Sprague–Dawley rats (190–210 g) were kept under standard housing conditions and fed ad libitum with regular laboratory chow. Pregnant rats were divided into three groups: in addition to absolute controls (no supplements at all), the vehicle-treated control animals received a small amount (650 mg) of liver pâté in pellet form once a day, whereas treated rats were given daily oral doses of RST (0.25 mg/kg body weight) mixed into pellets of liver pâté. Both groups received this liver pâté supplement (with or without RST) for 10 days, beginning on the 11th day of pregnancy (or until delivery). Feeding with the liver pâté was done individually, using forceps. Five breeding runs (4–6 pregnant rats each) provided the litters (6–12 pups from each mother) from which the independent experiments were performed. On postnatal day 1, the cerebral hemispheres of the absolute control, vehicle-treated control, and RST-treated rats were removed and either homogenized for Western blot analysis or embedded in paraffin for hematoxylin and eosin (H&E) staining and fluorescent immunohistochemistry/confocal microscopy.

### 4.2. Antibodies

The antibodies used in this study are listed in Tables S1 and S2. Several antibodies specific for the Lys methylation sites and the states of the H2A, H2B, H3, and H4 histone proteins were selected for the Western blot analyses and the fluorescent immunohistochemistry. The antibodies for cell-specific markers were used to detect neurons, astrocytes, oligodendrocytes, and microglial cells, as well as to check for possible changes in their ratios. The anti-Ki67 antibody was used to visualize the proliferating cells [64,65].

### 4.3. Histology

Rat brains were dissected quickly, fixed in 0.05 M phosphate-buffered saline (PBS) containing 4% formaldehyde, and then embedded in paraffin for H&E staining and immunohistochemistry. Paraffin-embedded sections were cut (6 µm thickness) on a microtome (Leica RM2235; Leica Mikrosysteme Vertrieb GmbH, Wetzlar, Germany), mounted on glass slides coated with (3-aminopropyl)triethoxysilane (Menzel, Darmstadt, Germany), and subsequently used for H&E staining and immunohistochemistry.

### 4.4. Fluorescent and Confocal Immunohistochemistry

The paraffin-embedded tissue sections were deparaffinized, rehydrated, and placed in a jar filled with 0.01 M citrate buffer (pH 6.0) containing 0.05% Tween-20, and then heated at 95 °C for 20 min. The sections were washed 3 × 10 min in 0.05 M PBS containing 0.05% Tween-20 and blocked in a 0.05 M PBS solution containing 0.05% Tween-20 and 5% normal goat serum (NGS) for 1 h at room temperature (RT). The sections were then incubated with primary antibodies in a 0.05 M PBS solution containing 0.05% Tween 20 and 5% NGS overnight at 4 °C. After washing (4 × 10 min in 0.05 M PBS containing 0.05% Tween-20), the primary antibodies were labeled with either Alexa 488- or Alexa 568–conjugated secondary antibodies (final dilution 1:1000; Invitrogen, Carlsbad, CA, USA) in a blocking solution for 3 h at RT. After 4 × 10 min washes in 0.05 M PBS containing 0.05% Tween-20, the cell nuclei were stained using a 2-[4-(aminoiminomethyl)phenyl]-1H-indole-6-carboximidamide hydrochloride (DAPI) solution (Thermo Fisher Scientific, Waltham, MA, USA). Digital images were captured on a Leica DMLB epifluorescence microscope using a Leica DFC7000 T CCD camera (Leica Microsystems CMS GmbH, Wetzlar, Germany) and the LAS X Application Suite X (Leica).

Select immuno-labeled sections were also examined with a confocal laser scanning microscope (Olympus Fluoview FV1000, Olympus Life Science Europa GmbH, Hamburg, Germany). Images (512 × 512 pixels) were captured along the *Z*-axis, with a distance of 0.5 μm between consecutive optical slices using the following microscope configuration: objective lens, UPLSAPO 60×; numerical aperture, 1.35; sampling speed, 4 μs/pixel; optical zoom, 2×; and scanning mode, sequential unidirectional. The excitation wavelengths were as follows: 405 nm (DAPI), 488 nm (Alexa Fluor 488), and 543 nm (Alexa Fluor 568). Z-stack images were prepared using 10–12 consecutive optical slices.

### 4.5. Western Blot Analyses

Brains from newborn rats were dissected, homogenized in 50 mM Tris-HCl (pH 7.5 at 4 °C) containing 150 mM NaCl, 2 μg/mL leupeptin, 1 μg/mL pepstatin, 2 mM phenylmethylsulfonyl fluoride, and 2 mM EDTA, and centrifuged at 14,000× *g* for 10 min. The supernatant was aliquoted, and the pellet was rehomogenized in 50 mM Tris-HCl (pH 7.5 at 4 °C) containing 150 mM NaCl, 0.1% Nonidet-P40, 0.1% cholic acid, 2 μg/mL leupeptin, 1 μg/mL pepstatin, 2 mM phenylmethylsulfonyl fluoride, and 2 mM EDTA. The samples were incubated on ice for 30 min and then aliquoted. The protein concentrations of these suspensions and the abovementioned supernatants were determined by the Lowry method [66]. For Western blot analyses, 15–30 μg of protein was separated on an SDS polyacrylamide gel (4–12% stacking gel/resolving gel), transferred onto a Hybond-ECL nitrocellulose membrane (Amersham Biosciences, Little Chalfont, Buckinghamshire, England), blocked for 1 h in 5% nonfat dry milk in Tris-buffered saline (TBS) containing 0.1% Tween-20, and incubated overnight with the appropriate primary antibodies as well as with those of the internal control (mouse anti-GAPDH monoclonal antibody). After five rinses in 0.1% TBS–Tween-20, the membranes were incubated for 1 h with the appropriate horseradish peroxidase-conjugated goat anti-rabbit or rat anti-mouse secondary antibodies and washed three times, as described above. The enhanced chemiluminescence method (ECL Plus Western blotting detection reagents; Amersham Biosciences) was used to reveal the immunoreactive bands, according to the manufacturer’s protocol. The exposure time and film development were optimized for each antibody.

### 4.6. Imaging and Statistical Analyses

Grayscale digital images of the immunoblots were acquired by scanning the autoradiographic films with a desktop scanner (Epson Perfection V750 PRO; Seiko Epson Corp., Suwa, Japan). The images were scanned and processed at identical settings to allow comparisons of the Western blots obtained from different samples. The bands were analyzed by densitometry via ImageJ (version 1.47; developed at the U.S. National Institutes of Health (Bethedsa, MD, USA) by W. Rasband, available at https://imagej.net/Downloads; accessed on 10 July 2013) [67]. The immunoreactive densities of equally loaded lanes were quantified, the samples were normalized to the densities of internal controls (GAPDH), and, for epigenetic studies, presented as % of the absolute controls or as % of controls.

All statistical comparisons were made using SigmaPlot software (v. 12.3, Systat Software, Inc., Chicago, IL, USA), and data were analyzed with one-way analysis of variance (ANOVA) or the Mann–Whitney rank–sum test. For the Western blots, values were presented as means ± standard errors of means (SEMs) from at least five immunoblots, each representing an independent newborn. A *p*-value of < 0.05 was considered significant.

## Data Availability

Data are contained within the article.

## Supplementary Materials

The following are available online at https://www.mdpi.com/1422-0067/22/7/3412/s1, Table S1: Primary and secondary antibodies used in immunohistochemistry, Table S2: Primary and secondary antibodies used in Western blots.

## Abbreviations

ANOVA	analysis of variance
CNS	central nervous system
CNPase	2′,3′-cyclic nucleotide 3′-phosphodiesterase
DAPI	2-[4-(aminoiminomethyl)phenyl]-1H-indole-6-carboximidamide hydrochloride
EAAT1	excitatory amino acid transporter 1 (also known as glutamate aspartate transporter 1 (GLAST-1))
GAPDH	glyceraldehyde 3-phosphate dehydrogenase (EC 1.2.1.12)
GFAP	glial fibrillary acidic protein
H	histone
HGM-CoA	3-hydroxy-3-methylglutaryl coenzyme A
H&E	hematoxylin and eosin
Iba1	ionized calcium-binding adaptor molecule 1
IgG	immunoglobulin G
Ki67	proliferation marker antigen identified by the monoclonal antibody Ki67
NeuN	anti-neuronal nuclei protein (hexaribonucleotide-binding protein-3)
PBS	phosphate-buffered saline
RST	rosuvastatin
RT	room temperature
SDS	sodium dodecyl sulfate
SEM	standard error of the mean
TBS	Tris-buffered saline
