# Supplementary material for: Epigenetic Consequences of in Utero Exposure to Rosuvastatin: Alteration of Histone Methylation Patterns in Newborn Rat Brains
Source: Int J Mol Sci. 2021 Mar 26;22(7):3412. doi: 10.3390/ijms22073412 (PMC8059142; doi:10.3390/ijms22073412)
Supplement: Supplementary file 1 [file ijms-22-03412-s001.zip › Table S1.docx]

Table S1. Primary and secondary antibodies used in immunohistochemistry

| Primary antibody, abbrev. name | | Primary antibody, full name | | Final dilution | | Company name | Secondary antibody with fluorochrome, full name | Company | Final dilution |
| --- | --- | --- | --- | --- | --- | --- | --- | --- | --- |
| NeuN | | Mouse anti-NeuN, monocl. ab. | | 1:100 | | Chemicon, Temecula, CA, USA | Alexa 488–conjugated anti-mouse IgG | Invitrogen, Carlsbad, CA, USA | 1:1000 |
| Iba1 | | Mouse anti-Iba1, monocl. ab. | | 1:250 | | Abcam, Cambridge, UK | Alexa Fluor 488 goat anti‐mouse IgG | Invitrogen, Carlsbad, CA, USA | 1:1000 |
| Iba1 | | Rabbit anti-Iba1, polycl. ab. | | 1:300 | | Abcam, Cambridge, UK | Alexa Fluor 568 goat anti‐rabbit IgG | Invitrogen, Carlsbad, CA, USA | 1:1000 |
| Ki-67 | | Rabbit anti Ki-67, polycl. ab. | | 1:400 | | Thermo Fisher Scientific, Inc., Waltham, MA, USA | Alexa Fluor 568 goat anti‐rabbit IgG | Invitrogen, Carlsbad, CA, USA | 1:1000 |
| GFAP | | Mouse anti-GFAP, monocl. ab. | | 1:100 | | Thermo Fisher Scientific, Inc., Waltham, MA, USA | Alexa Fluor 488 goat anti‐mouse IgG | Invitrogen, Carlsbad,  CA, USA | 1:1000 |
| CNPase | | Mouse anti-CNPase, monocle. ab. | | 1:500 | Abcam, Cambridge, UK | Alexa Fluor 488 goat anti‐mouse IgG | Invitrogen, Carlsbad, CA, USA | 1:1000 |  |
| H3K4me1 | | Rabbit anti-Histone H3 (mono methyl K4) polycl. ab. | | 1:500 | | Biorbyt, Cambridge, UK | Alexa Fluor 568 goat anti‐rabbit IgG | Invitrogen, Carlsbad, CA, USA | 1:1000 |
| H3K4me3 | | Rabbit anti-Histone H3 (tri methyl K4) polycl. ab. | | 1:500 | | Biorbyt, Cambridge, UK | Alexa Fluor 568 goat anti‐rabbit IgG | Invitrogen, Carlsbad, CA, USA | 1:1000 |
