# Supplementary material for: Epigenetic Consequences of in Utero Exposure to Rosuvastatin: Alteration of Histone Methylation Patterns in Newborn Rat Brains
Source: Int J Mol Sci. 2021 Mar 26;22(7):3412. doi: 10.3390/ijms22073412 (PMC8059142; doi:10.3390/ijms22073412)
Supplement: Supplementary file 1 [file ijms-22-03412-s001.zip › Table S2.docx]

Table S2. Primary and secondary antibodies used in Western blots

| Primary antibody, abbrev. name | Primary antibody, full name | Final dilution | Company name | Secondary antibody with fluorochrome, full name | Company | Final dilution |
| --- | --- | --- | --- | --- | --- | --- |
| H2AK118me1 | Rabbit anti-Histone H2A (mono methyl K118) monocl. ab. (EPR17488) | 1:1000 | Abcam, Cambridge, UK | Anti-rabbit IgG, peroxidase conjug. | Sigma, St. Louis, MO, USA | 1:2000 |
| H2BK5me1 | Rabbit anti-Histone H3B (mono methyl K5) polycl. ab. | 1:1000 | Abcam, Cambridge, UK | Anti-rabbit IgG, peroxidase conjug. | Sigma, St. Louis, MO, USA | 1:2000 |
| H3 | Rabbit anti-Histone H3 polycl. ab. | 1:1500 | Abcam, Cambridge, UK | Anti-rabbit IgG, peroxidase conjug. | Sigma, St. Louis, MO, USA | 1:2000 |
| H3K4me1 | Rabbit anti-Histone H3 (mono methyl K4) polycl. ab. | 1:1000 | Biorbyt, Cambridge, UK | Anti-rabbit IgG, peroxidase conjug. | Sigma, St. Louis, MO, USA | 1:2000 |
| H3K4me3 | Rabbit anti-Histone H3 (tri methyl K4) polycl. ab. | 1:1000 | Biorbyt, Cambridge, UK | Anti-rabbit IgG, peroxidase conjug. | Sigma, St. Louis, MO, USA | 1:2000 |
| H3K9me3 | Rabbit anti-Histone H3 (tri methyl K9) polycl. ab. | 1:1000 | Abcam, Cambridge, UK | Anti-rabbit IgG, peroxidase conjug. | Sigma, St. Louis, MO, USA | 1:2000 |
| H3K27me3 | Rabbit anti-Histone H3 (tri methyl K27) polycl. ab. | 1:1000 | Biorbyt, Cambridge, UK | Anti-rabbit IgG, peroxidase conjug. | Sigma, St. Louis, MO, USA | 1:2000 |
| H3K36me2 | Rabbit anti-Histone H3 (di methyl K36) monocl. ab. (EPR16994(2)) | 1:5000 | Abcam, Cambridge, UK | Anti-rabbit IgG, peroxidase conjug. | Sigma, St. Louis, MO, USA | 1:2000 |
| H4 | Rabbit anti-Histone H4 polycl. ab. | 1:1500 | Abcam, Cambridge, UK | Anti-rabbit IgG, peroxidase conjug. | Sigma, St. Louis, MO, USA | 1:2000 |
| H4K20me2 | Rabbit anti-Histone H4 (di methyl K20) polycl. ab. | 1:2000 | Abcam, Cambridge, UK | Anti-rabbit IgG, peroxidase conjug. | Sigma, St. Louis, MO, USA | 1:2000 |
| H4K20me3 | Rabbit anti-Histone H4 (tri methyl K20) polycl. ab. | 1:1000 | Abcam, Cambridge, UK | Anti-rabbit IgG, peroxidase conjug. | Sigma, St. Louis, MO, USA | 1:2000 |
| GAPDH | Mouse anti-GAPDH, monocl. ab., clone GAPDH-71.1 | 1:20000 | Sigma, St. Louis, MO, USA | Anti-mouse IgG, peroxidase conjug. | Sigma, St. Louis, MO, USA | 1:2000 |
| Iba1 | Rabbit anti-Iba1, polycl. ab. | 1:500 | FUJIFILM Wako, Osaka, Japan | Anti-rabbit IgG, peroxidase conjug. | Sigma, St. Louis, MO, USA | 1:2000 |
| Beta III Tubulin | Mouse anti-beta III Tubulin, monocl. ab., clone TU-20 | 1:500 | Abcam, Cambridge, UK | Anti-mouse IgG, peroxidase conjug. | Sigma, St. Louis, MO, USA | 1:2000 |
| EAAT1 | Mouse anti-EAAT1, monocl. ab. | 1:1000 | Abcam, Cambridge, UK | Anti-mouse IgG, peroxidase conjug. | Sigma, St. Louis, MO, USA | 1:2000 |
| CNPase | Mouse anti-CNPase, monocl. ab. | 1:250 | Abcam, Cambridge, UK | Anti-mouse IgG, peroxidase conjug. | Sigma, St. Louis, MO, USA | 1:2000 |
